# Angle imaging: Advances and challenges

**DOI:** 10.4103/0301-4738.73699

**Published:** 2011-01

**Authors:** Desmond T L Quek, Monisha E Nongpiur, Shamira A Perera, Tin Aung

**Affiliations:** 1 Singapore National Eye Centre, National University of Singapore; 2 Singapore Eye Research Institute, National University of Singapore; 3 Yong Loo Lin School of Medicine, National University of Singapore

**Keywords:** Anterior chamber angle imaging, anterior segment optical coherence tomography, EyeCam, gonioscopy, Scheimpflug photography, ultrasound biomicroscopy

## Abstract

Primary angle closure glaucoma (PACG) is a major form of glaucoma in large populous countries in East and South Asia. The high visual morbidity from PACG is related to the destructive nature of the asymptomatic form of the disease. Early detection of anatomically narrow angles is important and the subsequent prevention of visual loss from PACG depends on an accurate assessment of the anterior chamber angle (ACA). This review paper discusses the advantages and limitations of newer ACA imaging technologies, namely ultrasound biomicroscopy, Scheimpflug photography, anterior segment optical coherence tomography and EyeCam, highlighting the current clinical evidence comparing these devices with each other and with clinical dynamic indentation gonioscopy, the current reference standard.

## Angle Imaging: Advances and Challenges

Glaucoma is the second leading cause of blindness worldwide, affecting 60.5 million people in 2010, increasing to 79.6 million by 2020.[1] Asians will represent 47% of those with glaucoma and 87% of those with primary angle closure glaucoma (PACG). Bilateral blindness will be present in 3.9 million people with PACG in 2010, rising to 5.3 million people in 2020.[1] PACG has a mean annual age-and-sex-adjusted incidence of up to 8.3 per 100,000,[2] and is a major form of glaucoma in East and South Asia.[3–7] Aggressive and visually destructive, it is responsible for the majority of bilateral glaucoma blindness in Mongolia,[3] Singapore,[4] China[8] and India.[5] In China, an estimated 28 million have appositional angle closure, the anatomical trait predisposing to PACG, which blinds more people than open angle glaucoma in China.[7] The high prevalence of PACG in the populous nations like China and India ranks it as a major cause of significant visual morbidity on a global scale.

There exists a spectrum of stages in the PACG disease process. At the earliest stage (termed primary angle closure suspect, PACS), eyes have narrow or occludable angles without raised intraocular pressure (IOP) or glaucomatous optic neuropathy. Primary angle closure (PAC) is said to occur in eyes with narrow angles and the sequelae of apposition, peripheral anterior synechiae (PAS) and/or raised IOP but without glaucomatous optic neuropathy. PACG is reserved for cases of PAC with glaucomatous optic neuropathy. Although the natural history and clinical course of eyes with angle closure are not well established, PACS are anatomically predisposed and considered the “precursor” to PAC and PACG. It has been estimated that 22% of the eyes with PACS progress to PAC[9] and 28.5% progress from PAC to PACG over 5–10 years.[10] Prophylactic laser iridotomy performed as the first-line treatment for narrow angles may halt the progression of the angle closure process and prevent development of PACG,[11] but it is less effective in controlling IOP if optic nerve damage with PAS has already occurred.[12,13]

Many cases of PACG are asymptomatic and often present with severe to end-stage visual field loss at the time of the first presentation. The high visual morbidity from PACG is related to the destructive nature of the asymptomatic form of the disease.[14] Hence, early detection of anatomically narrow angles is important and the subsequent prevention of visual loss from PACG depends on an accurate assessment of the anterior chamber angle (ACA).

### Gonioscopy

Dynamic indentation gonioscopy is the current reference standard for assessing ACA structures and their configuration. The identification of regions of apposition of the iris to the trabecular meshwork enable the diagnosis of angle closure to be sought. However, gonioscopy is unfortunately plagued by subjectivity, with only moderate agreement reported among the observers.[3,4,15] The varying annotation of angle findings across different grading schemes,[16,17] varying gonioscopic findings with different gonioscopic lenses and the alteration of the angle configuration by light, placement of the lens and/or mechanical compression of the eye lead to significant variability in the assessments.[3,4,18–23] The definition of what constitutes an occludable angle also ranges from 180 to 270 degrees of angle, in which the trabecular meshwork is not visible.[24]

## Ultrasound biomicroscopy

Ultrasound biomicroscopy (UBM), a technique first developed in the 1990s, is an objective alternative for ACA assessment. Electric signals are converted, by a radiofrequency signal generator coupled to a piezoelectric transducer, into 50 MHz frequency ultrasonic sound waves, which are transmitted to the eye via saline solution that is held in a cup reservoir or within the end of a probe on which the transducer is mounted. These sound waves travel at different speeds through the eye as they encounter tissues of varying acoustic impedance and are reflected at differing time intervals. A computer system collates and magnifies these reflected sound waves, providing a high-resolution B scan image.

**Figure 1:**
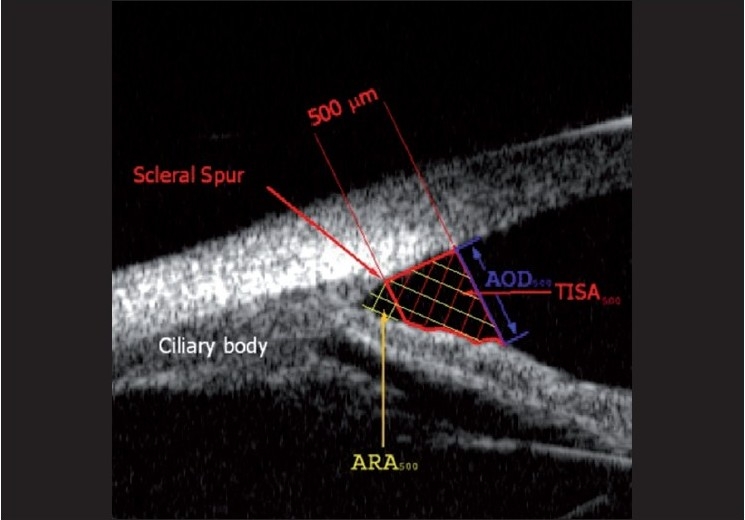
Angle parameters on ultrasound biomicroscopy, showing the trabeculo–iris space area at 500 µm (TISA_500_), angle recess area at 500 µm (ARA_500_) and angle opening distance

Studies comparing UBM to gonioscopy have found a high agreement between the two modalities when both are performed in a completely dark room.[25] UBM is sufficiently sensitive such that significant differences among the mean UBM measurements (angle-opening distances at 250 µm and 500 µm from the scleral spur and trabecular meshwork–ciliary process distance) of each angle grade estimated by gonioscopy can be detected [Fig. 1].[26] Although subjective gonioscopic assessment occasionally resulted in an overestimation of the angle width as compared with the UBM values in eyes with occludable angles,[27] angle dimensions measured by UBM correlated significantly with gonioscopy in general.[26]

UBM allows for the acquisition of real-time images, with lateral and axial resolutions of 50 µm and 25 µm, respectively.[28,29]

In addition, its ability to visualize posteriorly located structures such as the ciliary body, lens zonules and anterior choroid puts it at an advantage over other modalities, especially for the investigation of the mechanisms behind angle closure. This includes anterior rotation of the ciliary body in plateau iris, iridociliary masses causing secondary angle closure or choroidal effusions.[28,30] Additionally, UBM may also play a role in the evaluation of certain types of secondary glaucoma, such as pigment dispersion[31] (posteriorly bowed, causing iris pigment shaffing) and assessing for a tilted or subluxed lens in the exfoliation syndrome.[32]

**Figure 2:**
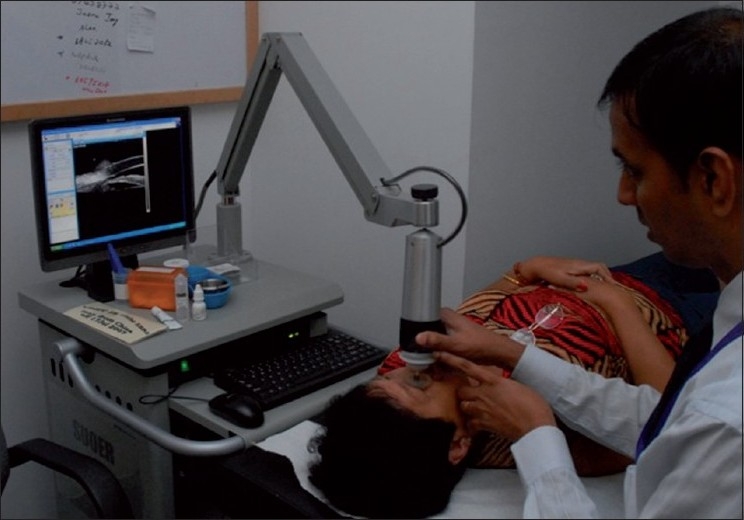
Ultrasound biomicroscopy procedure

When performing UBM, the requirement for a saline bath, through which sound waves are transmitted, necessitates contact with the eye, usually in the form of a scleral cup or a corneal probe [Fig. 2]. This introduces discomfort, the need for a supine position, risks of mechanical corneal abrasion and infection and the likelihood of angle distortion due to inadvertent indentation.[33] Optimal UBM imaging requires a skilled operator and cooperative subject and, even then, the process can be time consuming.

### Scheimpflug photography

The Scheimpflug principle describes the change in focal plane that occurs when the film plane is tilted, such that the focal, lens and film planes are not parallel, shifting the plane of sharp focus to the intersection point of the film and lens planes and allowing slit images of the anterior segment of the eye that retain depth to be obtained. Commercial devices based on this principle now take up to 50 images in 2 s, using a rotating camera, which are reconstructed into a 3-dimensional image, enabling a rapid assessment of the anterior chamber. Semi-automated analysis of angle width requires the user to determine the iris plane and plane of corneal curvature by placing up to 10 marks on the corneal endothelium, from which the angle width is measured. Although subjective, this fast and non-contact method of ACA assessment has been previously reported to be highly reproducible, at least in eyes with open angles.[34–38]

Scheimpflug photographic techniques, however, have not been documented to reliably image a variety of angle configurations. In addition, the ACA cannot be entirely visualized and only the angle approach can be photographed as light is unable to penetrate to the angle recess. User definition of the iris plane necessarily uses a straight line to describe a curved plane, leading to inaccuracies in angle width measurement. Comparing ACA width measurements using Scheimpflug photography and UBM revealed only moderate correlation,[39] with Scheimpflug images being of a much lower resolution. In addition, one study found that angle measurements from Scheimpflug images were less sensitive to changes in illumination compared with those obtained using UBM.[40] In a recent study, Scheimpflug photography was reported to provide insufficient detail of the angle for assessment of angle anatomy, with limited agreement existing between gonioscopy, Scheimpflug photography and UBM.[41]

### Anterior segment optical coherence tomography

Anterior segment optical coherence tomography (AS-OCT) is a rapid, non-contact imaging device that acquires high-resolution cross-sectional images of the anterior segment structures and allows for their objective and quantitative evaluation. This imaging technology uses low-coherence interferometry to measure the delay and intensity of light reflected from tissue structures and comparing it with light that has traversed a known reference path length by using a Michelson-type interferometer.[42] The A-scans in this time-domain OCT technology are produced by varying the position of the reference mirror. Although this principle was originally employed for the retinal OCT using light of wavelength 830 nm,[43,44] it was later modified and refined to image the anterior segment[45] by altering the light to a longer wavelength of 1310 nm.[46] This increases the depth of penetration by reducing the amount of light scattered by the sclera and limbus, allowing for visualization of the ACA morphology in greater detail. In addition, the 1310 nm light incident on the cornea is strongly absorbed by water in the ocular media, with only 10% reaching the retina.[47] This enables the AS-OCT to utilize higher power, enhancing imaging speed and eliminating motion artefacts.

### Visante and slit-lamp AS-OCT

**Figure 3:**
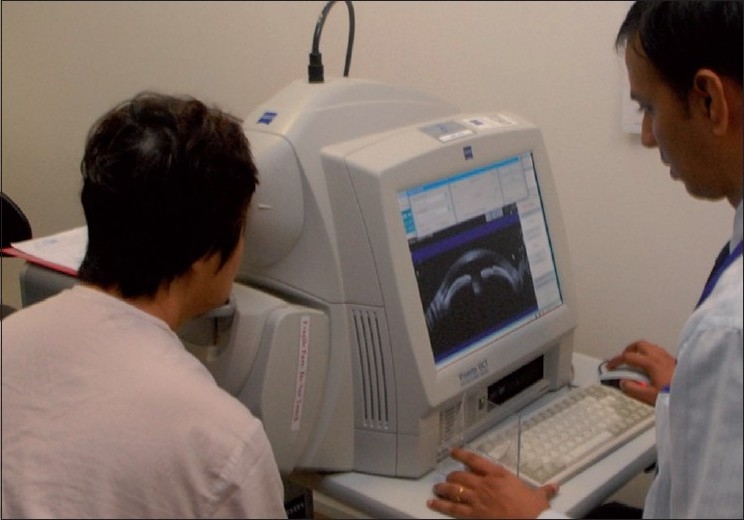
Visante anterior segment optical coherence tomography procedure

The Visante AS-OCT (Carl Zeiss Meditec Inc., Dublin, CA, USA) obtains scans at a rate of 2000 A-scans per second, with an axial and transverse resolution of 18 µm and 60 µm, respectively. It requires minimal experience for image acquisition [Fig. 3]. Slit-lamp OCT (SL-OCT) (Heildelberg Engineering, Heildelberg, Germany) is the other commercially available AS-OCT device that is incorporated into a modified slit-lamp biomicroscopy system. Compared with the Visante OCT, the SL-OCT has a slower image acquisition speed and a lower axial and transverse resolution of <25 µm and 20–100 µm, respectively. Furthermore, the SL-OCT requires manual rotation of the scanning beam.

The AS-OCT devices provide anterior segment, angle and corneal scans and pachymetry maps and can also be used to calculate the depth, width and angle of the anterior chamber. Customized software devices have also been developed to quantitatively assess, in greater detail, angle parameters, namely trabeculo-iris space area (TISA), angle recess area (ARA) and angle opening distance (AOD).

**Figure 4:**
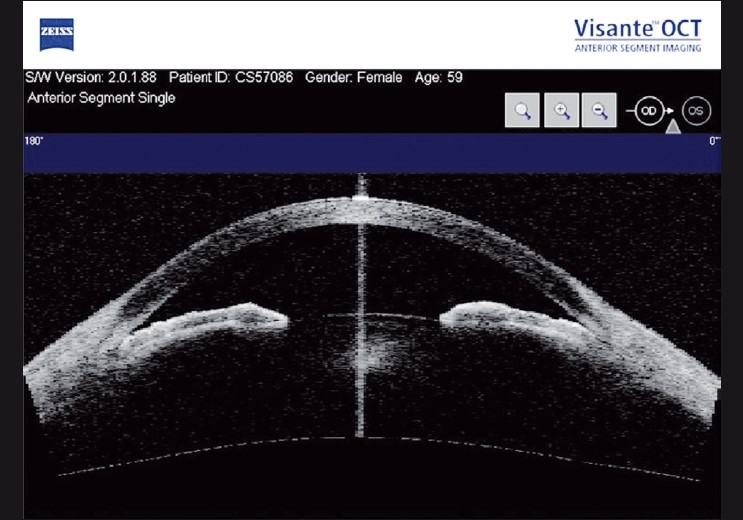
Visante anterior segment optical coherence tomography image showing closed angles

Comparison studies between Visante AS-OCT and gonioscopy found the AS-OCT detected greater closed angles than gonioscopy [Fig. 4], particularly in the superior and inferior quadrants.[48,49] Using gonioscopy as the reference standard, the sensitivity and specificity of AS-OCT to identify angle closure were 98% and 55.4%, respectively, using a definition of one or more quadrants of non-visibility of the trabecular meshwork.[48] Several explanations have been suggested for the disparate findings between gonioscopy and AS-OCT. Inadvertent pressure on the globe and too much exposure of the pupil to visible light during gonioscopy may alter the configuration of the angle, leading to spurious widening of the angle. Another reason could be a difference in the definition and description of the landmarks used to define angle closure. On gonioscopy, angle closure was defined as the apposition between the iris and the posterior trabecular meshwork, whereas on the AS-OCT, it was defined as any contact between the iris and the angle structures anterior to the sclera spur.

Comparison of ACA dimensions by SL-OCT and Visante AS-OCT found a poor correlation, suggesting that angle measurements obtained by the two devices cannot be used interchangeably.[50] The authors attributed the poor agreement to differences in the choice of refractive indices in the calculation of anterior segment dimensions, differing algorithms for image dewarping, software for image analysis, exact scan location and use of internal fixation in Visante OCT and external fixation in SL-OCT. Another study comparing the two AS-OCT devices found that both detected more closed angles than gonioscopy.[51] However, there was a better agreement between SL-OCT and gonioscopy, which can be attributed to the use of visible light during both examinations. The study also found discrepancies in the ACA quantification with each device, confirming the findings of Leung *et al*.[50] that the measurements are not interchangeable.

In a comparison between low-resolution (20 µm) and high-resolution (8 µm) AS-OCT, Wang *et al*. found that higher-resolution OCT produced larger-angle width measurements, which they attributed to the different image-processing algorithms, where dewarping procedures were not implemented for the high-resolution images.[52] Moreover, sclera spur location was more accurately determined in the images obtained by the higher-resolution mode.

The major advantages of the AS-OCT devices include ease of operation and rapidity of image acquisition. The non-contact method eliminates patient discomfort and inadvertent compression of the globe, which is especially useful in the immediate post-operative period or after trauma. The incorporation of automated analysis software allows for rapid estimation of the various anterior segment parameters, including corneal thickness, anterior chamber depth and ACA indices. In addition, customized image analysis software can be also be used to quantify other angle parameters.

The main disadvantage of these devices is the inability to distinctly detect and measure structures posterior to the iris as well as peripheral anterior synechiae. Currently available software analysis programs require the manual localization of the scleral spur, which can at times be difficult, especially in closed angles or where there is a smooth transition from cornea to sclera. Sakata *et al*. found that the sclera spur could not be detected in approximately 30% of the ACA quadrants, this problem being worse in the superior and inferior quadrants.[49] While gonioscopy allows concurrent and dynamic visualization of the entire angle quadrant, AS-OCT images should only be interpreted for the particular section of the ACAs scanned. Lastly, the high cost of these devices may be a limiting factor for their use in routine clinical set-up or screening purposes.

### Spectral domain OCT

**Figure 5:**
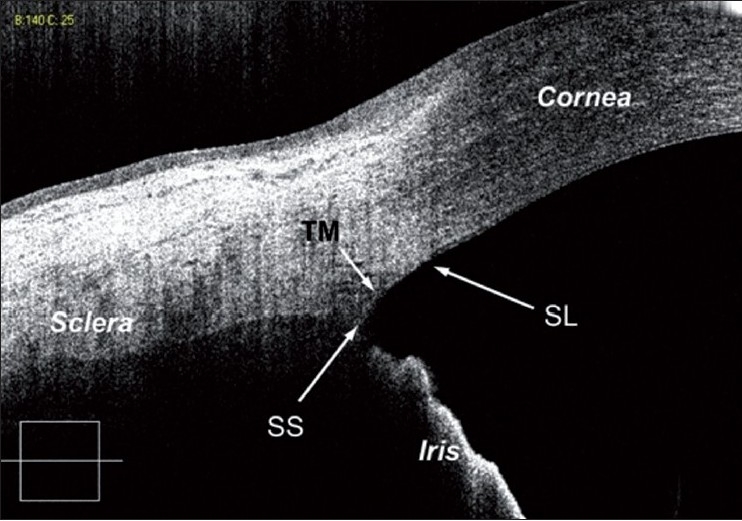
High-resolution spectral domain optical coherence tomography image showing angle structures: trabecular meshwork, Schwalbe’s line and scleral spur

Fourier or Spectral domain OCT (SD-OCT) differs from time domain OCT (TD-OCT) by utilizing light of shorter wavelength (830 nm) and having a fixed reference mirror, which allows higher scanning speed and more images to be taken in a single pass.[53] It scans at a rate of 26,000 A-scans per second, producing detailed cross-sectional images of structures at an axial resolution of 5 µm and a transverse resolution of 15 µm [Fig. 5]. However, the shorter wavelength of the SD-OCT reduces the depth of penetration of the anterior segment structures, making it useful for imaging the corneal region and less useful for the iris and more posterior areas. The RTVue (Optovue Inc.,Fremont, CA, USA) is an SD-OCT system that can be used for either retinal or anterior segment imaging (when used with a corneal adaptor module, CAM).

The Cirrus high-definition OCT (HD-OCT) 4.0 (Cirrus; Carl Zeiss Meditec Inc.) is used for *in vivo* viewing, axial cross-sectional and three-dimensional imaging and measurement of the anterior and posterior ocular structures.

Stehouwer and colleagues recently described a novel technique of integrating a combined anterior and posterior segment SD-OCT (SLSCAN-1) onto a slit-lamp, with the aim of improved efficiency in the clinical evaluation of a patient.[54] With regards to anterior segment scan, the authors acknowledge that the images were not comparable to the commercially available TD-OCT devices.

In a recent study, Wong and colleagues modified a commercially available SD-OCT device with a 60 diopter lens to acquire high-resolution images of the ACA.[55] In addition to the sclera spur, they were able to identify new angle imaging landmarks such as the Schwalbe’s line in 93.3% and trabecular meshwork in 62.2% of the images. Although the device also showed a good correlation with gonioscopy findings (kappa = 0.65), better than that obtained by AS-OCT (AC1 = 0.35–0.47),[51] it detected fewer closed angles compared with gonioscopy. Wylegala and colleagues compared anterior segment imaging and measurements obtained by the TD- and SD-ASOCT systems.[56] Quantitatively, they found no statistical difference between the mean TISA and AOD values measured by the two devices.

The SD-OCT devices seem to allow better structural delineation and visualization of novel ACA landmarks such as Schwalbe’s line, trabecular meshwork and Schlemm’s canal.[55,56]

## EyeCam^™^

The EyeCam^™^ (Clarity Medical Systems, Pleasanton, CA, USA) is a new technology originally designed to yield wide-field photographs of the pediatric fundus for the diagnosis and management of posterior segment diseases.[57] With modifications in the optical technique and the inclusion of a 130 degree lens, the device can be used to visualize angle structures in a manner similar to direct gonioscopy.

**Figure 6:**
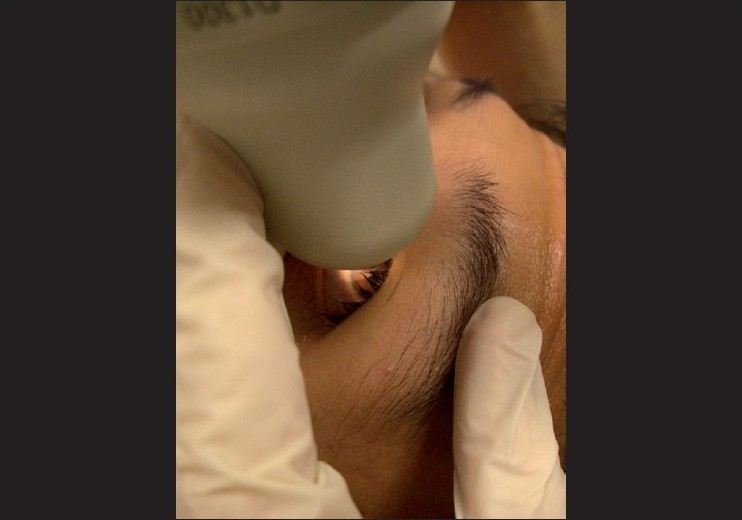
EyeCam procedure

For imaging, patients are positioned supine and the lens probe is placed on a coupling gel without direct contact onto the cornea, minimizing alteration of angle configuration due to compression artefact and causing less discomfort than gonioscopy [Fig. 6]. To image a particular angle quadrant, the patient is instructed to look in the direction of that angle. The probe is positioned at the opposite limbus to the angle being photographed and light from the fiber optic probe is directed toward the angle of interest and then tilted downward, to bring the angle structures into view while minimizing pupillary constriction.

**Figure 7:**
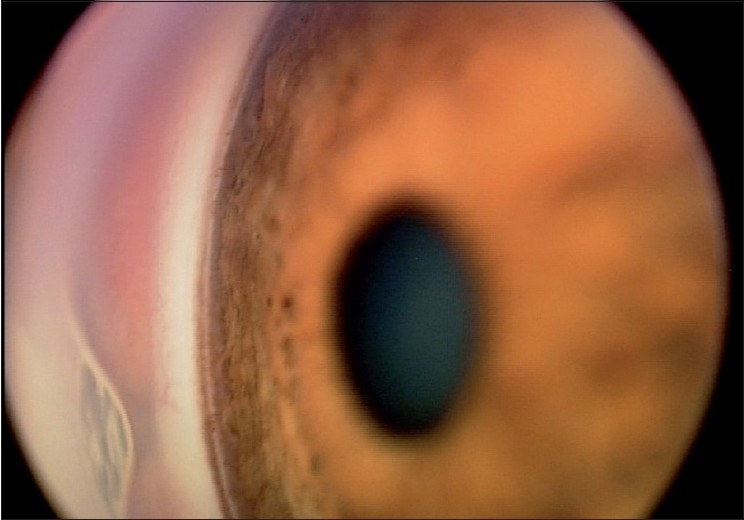
Image of an open angle obtained using EyeCam, detailing, clearly, the Schwalbe’s line, pigmented trabecular meshwork, scleral spur and iris processes

The EyeCam^™^ is thus a new and objective way of documenting angle configuration, using a photographic method similar to goniophotography. The images produced are easy for clinicians to interpret as the angle appears similar to what is seen during gonioscopy [Fig. 7]. Images recorded by the EyeCam^™^ can be saved on a computer thus allowing comparisons to be made over time. Such “goniographic” documentation by EyeCam^™^ allows for monitoring of angle changes over time, tracking of angle changes with disease progression as well as treatment effects[58] and use as patient education tools.

A preliminary study comparing EyeCam^™^ “gonio-graphy” with conventional gonioscopy in 60 eyes, with angles ranging from a Shaffer grading of 0 to 4 on clinical gonioscopy, demonstrated that results from the EyeCam^™^ are accurate and reliable.[59] In another study comparing these two modalities, Perera *et al*. (2009, in press) found that the agreement between EyeCam^™^ and gonioscopy in detecting closed quadrants in the superior, inferior, nasal and temporal quadrants based on AC1 statistics was 0.73, 0.75, 0.76 and 0.72, respectively. EyeCam^™^ had 76% sensitivity and 81% specificity for detecting eyes with angle closure using the two-quadrant definition of angle closure to categorize each eye.

Although the EyeCam^™^ is as yet unable to provide quantitative measurements of anterior chamber depth like the AS-OCT or UBM, it provides a 360-degree visualization of the entire ACA compared with the ASOCT and UBM, which provide only cross-sectional views.

The device has some limitations: imaging of the ACA using EyeCam^™^ takes longer than gonioscopy (about 5–10 min per eye). The device is more expensive than gonioscopy and additional space is required for supine examination. It is not known if supine positioning would widen the angle due to the effect of gravity on the lens–iris diaphragm. The light source from the EyeCam^™^, delivered via a fiber optic cable, may cause pupil constriction, artificially altering ACA configuration. Unlike with dynamic gonioscopy, it is difficult to discern the presence of PAS due to the inability to indent the angle. Similarly, determination of iris configuration is difficult with the two-dimensional EyeCam^™^ images. Reproducibility may also be compromised as, with repeat imaging, each photograph may be slightly rotated and images may not be obtained over the exact same location, unless certain landmarks on the iris are used as anchors.

## Conclusions

New methods of imaging the angle have been introduced, offering advantages of being more objective, reproducible and non-contact, rapid image requisition and storage, quantitative analysis and the ability for anterior segment imaging despite corneal opacities results in their easy incorporation into clinical practice and research. While none of these new devices, singly, can replace conventional slit-lamp biomicroscopy and gonioscopy, these new techniques of anterior segment and ACA imaging are useful in complementing clinical practice, particularly when gonioscopy is difficult. While attempting to address the shortfalls of gonioscopy, these devices are not without their own limitations and, until the ultimate imaging tool is invented, clinical dynamic indentation gonioscopy remains the current reference standard.
